# Iron-laden blasts in refractory acute myeloid leukemia

**DOI:** 10.1007/s12308-023-00555-6

**Published:** 2023-07-29

**Authors:** Christopher Vossen, Roeun Im, Pedro Horna

**Affiliations:** Department of Laboratory Medicine and Pathology, Division of Hematopathology, Mayo Clinic, 200 1st St SW, MN 55905 Rochester, USA

**Keywords:** Acute myeloid leukemia, Green cytoplasmic granules, Hemosiderin-laden blasts

## Abstract

We report a case of refractory acute myeloid leukemia with *DEK-NUP214* rearrangement showing circulating monocytic blasts with abundant green cytoplasmic granules on the Wright-Giemsa stain. The granules were strongly positive for Perls’ Prussian blue, consistent with hemosiderin deposits. This previously unreported finding is suggestive of siderophage activity by leukemic blasts, likely associated with monocytic differentiation.

A 16-year-old adolescent girl was referred to our hospital with an eight-month history of refractory acute myeloid leukemia with t(6;9)(p23;q34.1) *DEK-NUP214* rearrangement. During the next four months, different chemotherapeutic approaches were tried, with no response on subsequent bone marrow biopsies. The patient progressively deteriorated and was transferred to the intensive care unit, where therapy with imatinib and sirolimus was attempted as salvage therapy.

A complete blood count showed hemoglobin of 6.2 g/dL, MCV 89.6 fL, white blood cells 7.0 × 10^9^/L, and platelets 52 × 10^9^/L. A Wright-Giemsa peripheral blood smear demonstrated 77% circulating blasts with overt monocytic differentiation and large green cytoplasmic granules detectable in approximately 15% of leukemic forms (Fig. 1A, B). Cytochemical staining revealed strong positivity with Perls’ Prussian blue (Fig. 1C, D), consistent with overt hemosiderin deposits within the cytoplasm of the monocytic leukemic blasts.

**Fig. 1:**
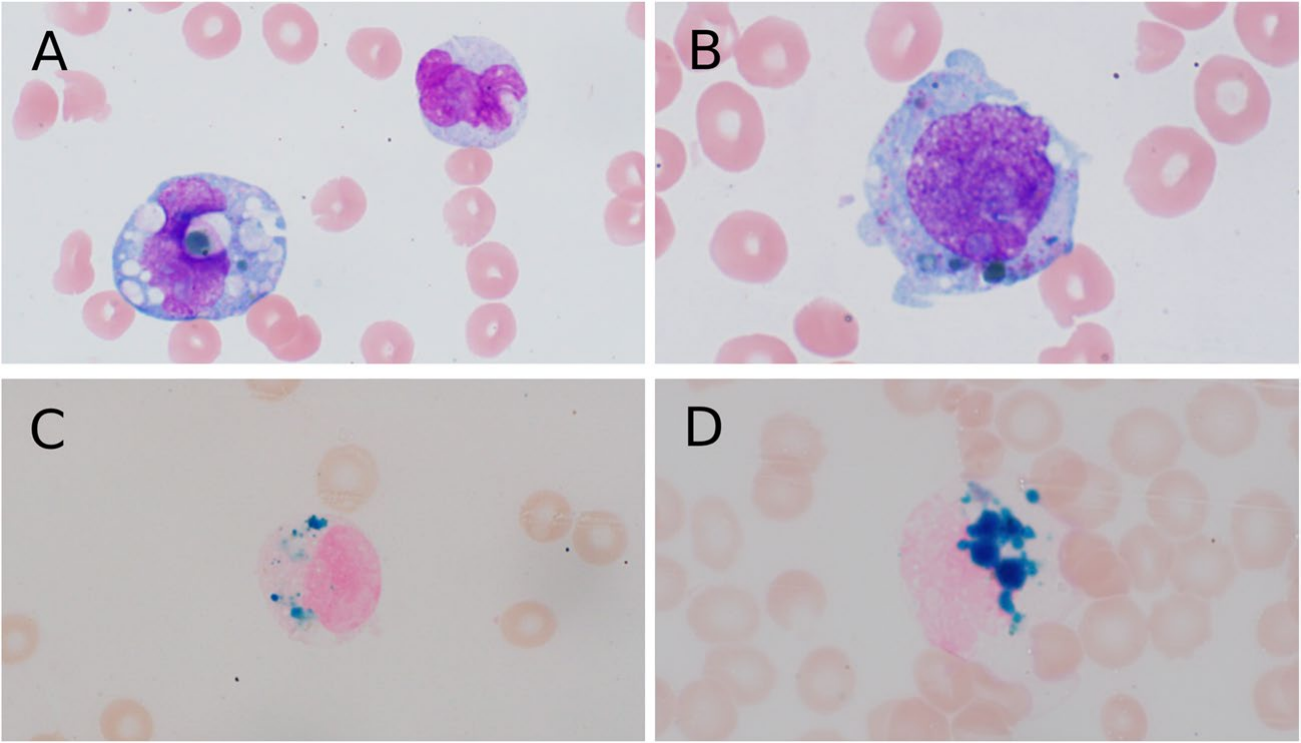
**A**, **B** The Wright-Giemsa stain of peripheral blood smear showing blasts with green cytoplasmic granules. **C**,** D** Perl’s Prussian blue stain of peripheral blood showing hemosiderin deposits in the cytoplasm of blasts

Hemosiderin deposits are cytoplasmic iron-storage complexes comprised of digested ferritin and lysosomes. They accumulate in benign macrophages after digestion of heme molecules (e.g., secondary to hemorrhage and erythrophagocytosis) [1] or in inflammatory states where secretion of hepatic hepcidin induces degradation of the macrophage iron exporter ferroportin and subsequent accumulation of cytoplasmic iron [2]. While the mechanism for leukemic hemosiderin accumulation, in this case, is unknown, the overt monocytic differentiation suggests a similar process to that of benign macrophages in response to inflammation. Previous peripheral blood smears and bone marrow biopsies demonstrated no evidence of erythrophagocytic blasts prior to the discovery of this finding. Unfortunately, 2 days after the discovery of these inclusions, the patient passed away.
